# Increased Atherosclerosis in Mice Deficient in Perilipin1

**DOI:** 10.1186/1476-511X-10-169

**Published:** 2011-09-24

**Authors:** Dominique Langlois, Fabien Forcheron, Jacques-Yuan Li, Peggy del Carmine, Samia Neggazi, Michel Beylot

**Affiliations:** 1 ERI22-EA4173, Faculté Rockefeller, University C Bernard Lyon1, 8 av Rockefeller, Lyon, 69008, France; 2 AniRA-ANIPHY, Faculté Rockefeller, University C Bernard Lyon1, 8 av Rockefeller, Lyon, 69008, France

**Keywords:** perilipin1, atherosclerosis, lipids

## Abstract

**Background:**

Perilipin1, a lipid droplet associated protein has an important role in the regulation of lipolysis and lipid storage in adipocytes. Perilipin1 is also expressed in foam cells of atheroma plaques and could therefore play a role in the accumulation of lipids in arterial wall and in the development of atherosclerosis. The aim of the study was to investigate this possible role of perilipin1 in atherogenesis.

**Methods:**

Mice deficient in perilipin1 (*Plin1-/-) *were crossed with *Ldlr-/- *mice. *Ldlr-/- *and *Plin1-/- Ldlr-/- *mice received an atherogenic diet during 10 or 20 weeks. Blood pressure and plasma lipids concentrations were measured. Aortas were collected at the end of the atherogenic diet periods for quantification of atheroma lesions (*en face *method), histological and immunohistological studies

**Results:**

*Ldlr-/- *and *Plin1-/- Ldlr-/- *mice had comparable blood pressure and plasma lipids levels. *Plin1-/- Ldlr-/- *mice had a lower body weight and decreased adiposity. The atherosclerotic lesion area in *Plin1-/-Ldlr-/- *mice was moderately increased after 10 weeks of atherogenic diet (ns) and significantly higher after 20 weeks (p < 0.01). Histology of atheroma plaques was comparable with no sign of increased inflammation in *Plin1-/- Ldlr-/- *mice.

**Conclusion:**

Perilipin1 ablation in mice results in increased atherosclerosis independently of modifications of risk factors such as raised blood pressure or plasma lipids levels. These data strongly support an atheroprotective role for perilipin1.

## Introduction

A hallmark of atherosclerosis is the accumulation of free (FC) and esterified (EC) cholesterol in macrophages and smooth muscular cells transforming them in foam cells [1]. Such accumulation depends on the balance between the uptake of cholesterol-rich lipoprotein through scavenger receptors [2] and the efflux of free cholesterol controled by the transporters ABCA1 and ABCG1 [3] and to a lesser extent by SR-B1 (or CLA-1) [4,5]. This accumulation depends also on the intra-cellular metabolism of cholesterol, particularly the balance between its free and esterified forms. CE taken up by cells is hydrolyzed in lysosomes to FC that is then directed to various cell membranes by the protein NPC1 [6]. Excessive accumulation of FC has toxic effects on cells [7] and FC must be either by removed through efflux to extra-cellular acceptors or esterified. CE is then stored in lipid droplets and can be removed from cells only after hydrolysis to FC by a cholesterol ester hydrolase, whose nature is still debated [8-10].

Storage of lipids droplets in cells accumulating triacylglycerols (adipocytes, hepatocytes) or EC (steroidogenic cells) is also dependent in part of specific proteins surrounding these droplets and belonging to the PERILIPIN family (previously named PAT family) [11], particularly perilipin1 (previously perilipin) and perilipin2 (previously adipophilin or ADRP). Perilipin2 is present in all cells storing lipids [12]. Its expression is increased during incubation of macrophages with oxidized LDL [13,14]. It is expressed in foam cells of atherosclerotic plaques [14]. Its overexpression in THP-1 macrophages enhances lipid accumulation [15] whereas its invalidation protects against atherosclerosis [16]. Therefore perilipin2 is clearly involved in atheroma. Perilipin1 has at least three different forms, perilipin1 A, B and C, resulting from alternative splicing of a common premessenger RNA [17]. Perilipin1 A and B are present in adipocytes, the A form being largely predominant. Perilipin1 C is found in steroidogenic cells. In adipocytes, perilipin1 opposes in the basal state hydrolysis of triacylglycerols. β-adrenergic agents phosphorylate perilipins1 on specific serine sites and phosphorylated perilipins 1 allow phosphorylated HSL to hydrolyze TG. Perilipins1 are thus implicated in the regulation of basal and stimulated lipolysis. Perilipin1 A is expressed in macrophages [18-21] and vascular smooth muscular cells [18], in arterial wall [18] and is overexpressed in atheroma plaques [18,22], particularly unstable plaques [22]. Perilipin1 could therefore be implicated in the development of atherosclerosis by controling the hydrolysis of stored EC. The overexpression of perilipin1 in atheroma plaque could favour the accumulation of cholesterol and promote the development of atheroma. However, perilipin1 could also shift the balance between FC and EC toward EC and help to prevent excessive accumulation of FC. Since excess FC is toxic for cells and plays a role in the evolution of plaques toward instability [7,23], perilipin1 could have on the contrary a protective role. In a first step to clarify this issue and to determine the role, if any, of perilipin1 in atheroma, we investigated the effect of perilipin1 invalidation on the development of atheroma in an experimental model, the *Ldlr *^-/- ^mouse.

## Methods

*Plin1*^*-/- *^mice were a gift from I Tabas (Columbia University, NY, USA). These mice are from the strain generated and described by Martinez-Botas et al [24] and are on a C57BL/6 background. *Ldlr*^-/- ^mice (C57BL/6 background) were from the Jackson Laboratory (Bar Harbor, ME, USA). *Plin1*^*-/- *^and *Ldlr*^-/- ^mice were crossed to obtain *Plin*^*+/-*^*Ldlr *^+/- ^mice, which were intercrossed to obtain *Ldlr*^-/- ^and *Plin1*^*-/- *^*Ldlr*^-/- ^mice. All mice were housed in an animal facility with controlled temperature (21-23°C) and lighting (light on 07:00, light off 19:00) and had free access to food and water. Male mice were used for the experiments. These mice received, starting at 8 weeks of age, an atherogenic diet (38.4% fat, 0.15% cholesterol, U8958 version 52 from SAFE, Augy, France) and were investigated after 10 (8 *Ldlr*^-/-^, 13 *Plin1*^*-/- *^*Ldlr*^-/- ^mice) or 20 weeks (13 *Ldlr*^-/-^, 30 *Plin1*^*-/- *^*Ldlr*^-/- ^mice) of atherogenic diet. This study was approved by the Ethical Committee of the University Cl Bernard of Lyon.

Blood pressure and heart rate were measured in 20 weeks old mice (8 *Ldlr*^-/-^and 16 *Plin1*^*-/- *^*Ldlr*^-/- ^mice) by the tail-cuff method (Visitech 2000 series II) after acclimation to restrain and tail-cuff inflation. For quantification of atheroma mice were anaesthetized with pentobarbital. Blood was collected from inferior vena cava and plasma separated by centrifugation for enzymatic determination (Biomerieux, Lyon, France) of total cholesterol and of triacylglycerols concentrations. Aortas were dissected from aortic root to iliac bifurcation, carefully cleaned form periarterial adipose tissue, pinned on silicon dishes and stained for lipid deposits with Red Sudan IV (*en face *method). Red Sudan IV positive areas were quantified using Image J software and expressed as the percentage of total aorta area. For histological studies some aortas of 20 weeks atherogenic diet fed mice were rinced with PBS and fixed in phosphate buffered formalin, carefully dissected, dehydrated and embedded in paraffin before sectioning. Haematoxylin-eosin and Verhoeff (elastic fibres) stainings were performed as well as immunocytology for macrophages (anti-Mac3 antibodies), lymphocytes (anti-CD3 antibodies) and smooth muscular cells (anti α-SMA antibodies) [25]. For measurements of mRNA concentrations aortas of 20 weeks atherogenic diet fed mice (5 *Ldlr*^-/-^and 5 *Plin1*^*-/- *^*Ldlr*^-/-^) were removed, flushed with cold isotonic saline, carefully cleaned of perivascular adipose tissue and flash frozen in liquid nitrogen before storage at -80°C until analysis. Total RNAs were purified using TRIZOL^R ^protocol (Invitrogen, Cergy-Pontoise, France) with the addition of a DNase treatment. Concentrations, intergrity and purity were verified. For measurements of individual mRNA levels (IL-6, IL1-β, TNFα, MCP-1, SR-A, ABCA1, ABCG1), total RNA was reverse transcripted using Superscript II (Invitrogen) and random hexamers. Real time PCR was performed in a MyIQ thermal cycler (Biorad, Marnes La Coquette, France) using iQ SYBR green Supermix (Biorad). Samples were run along with dilutions of known amounts of target sequence for quantification of initial cDNA copies. Results were calculated as the target over 18S RNA concentration ratio (ng/μg). Primer sequences are shown in table 1.

**Table 1:** Primers used for determinations of mRNAs concentration

	Forward primer	Reverse primer
IL-6	GCTGGAGTCACAGAAGG	TAGATGAGCCGTTTGGA

IL-1β	AGTCCCAGTGTTCYYGG	GCTGGACTGTTTCTAATGC

TNFα	GCCACCACGCTCTTCTG	GCTCACTGTTCGGACATCG

MCP-1	ACAACCACCTCAAGCAC	CAATACCATAAGGGAAAGT

ABCA1	TCCTGTGCCATTTATTC	GTTACTTAGTGGTCCTTCTT

ABCG1	ATGAATCAGCGAATGTTG	GTTCTAATGGGTGCCTCT

SR-A	CATCACCAACGACCTCAG	TGTCCAGTAAGCCCTCT

18S	TGAGGCCATGATTAAGAGGG	AGTCGGCATCGTTTATGGTC

Results are shown as individual values or as means ± sem. Comparisons between groups were performed by Student t test for non paired values or by Mann-Whitney test using GraphPad Prism (version 5.03). P < 0.05 was considered as indicating a significant difference.

## Results

**Figure 1:**
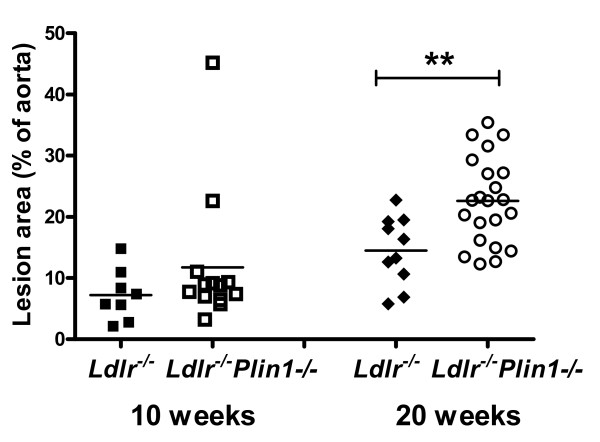
**Quantification of atheroma in aortas of *Ldlr***^***-/-***^**and *Ldlr***^***-/- ***^***Plin1***^***-/-***^**mice after 10 and 20 weeks of atherogenic diet**. Quantification was performed with staining of lipid deposits by Red Sudan IV (*en face *method). Results are shown as individual values and means. ** p < 0.01 vs *Ldlr*^*-/- *^mice.

**Figure 2:**
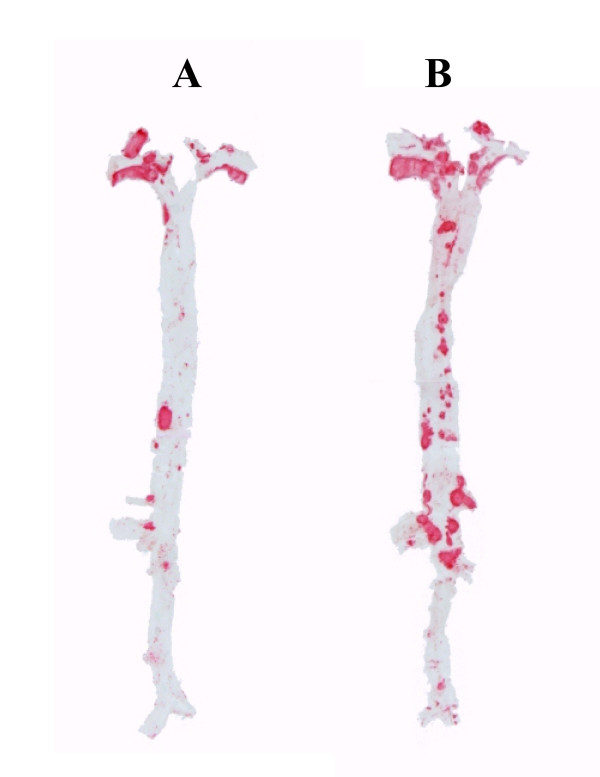
**Representative example of atheroma lesions in aorta isolated from *Ldlr***^***-/- ***^***Plin1***^***-/-***^**(right aorta, B) and *Ldlr***^***-/-***^**(left aorta, A) mice**. Staining with Red Sudan IV.

**Figure 3:**
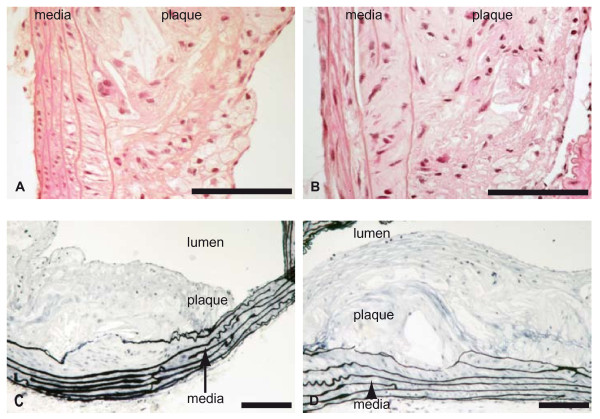
**Atherosclerosis in *Ldlr-/- *(panels A and C) and *Plin-/- Ldlr-/- *(panels B and D) mice**. Haematoxylin-eosin (panels A and B) and Verhoeff (panels C and D) stainings were performed on paraffin sections of aortas. Representative sections of atheroma plaques are shown. Magnification: ×10 (Ve rhoeff) ×20 (Haematoxylin-eosin). Scale: 100 μm.

**Figure 4:**
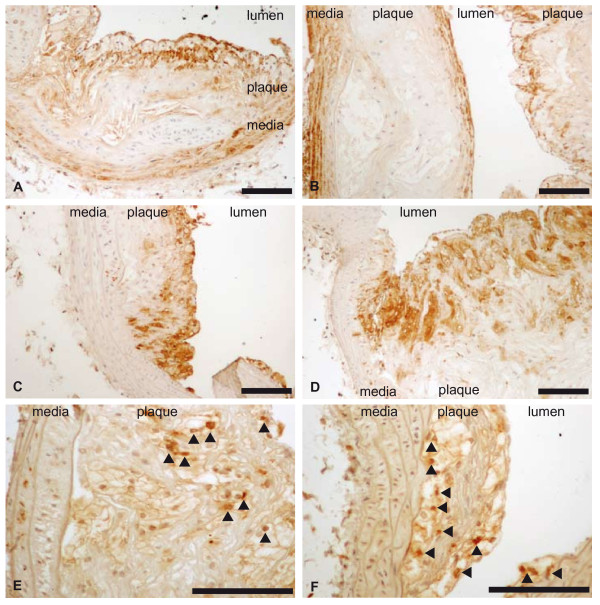
**Immunocytology of atheroma plaques of *Ldlr-/- *(panels A, C, E) and *Plin-/- Ldlr-/- *(panels B, D, F) mice**. Specific staining was performed with anti-Mac3 (macrophages, panels C and D), anti-CD3 (lymphocytes panel E and F) and anti-αSMA (smooth muscular cells, panel A and B) antibodies. Representative sections of atheroma plaques are shown. Scale: 100 μm.

Plasma cholesterol levels were high in both *Plin1*^*-/- *^*Ldlr*^-/- ^and *Ldlr*^-/- ^mice but did not differ between the two groups of mice (table 2). Plasma triacylglycerols levels were comparable. Systolic blood pressure (111.5 ± 3.1 vs 116.5 ± 6.6 mmHg in *Ldlr*^-/- ^mice) and heart rate (547 ± 25 vs 515 ± 23 b/min) were also comparable. *Plin1*^*-/- *^*Ldlr*^-/- ^had a slightly lower body weight (10 weeks: 25.2 ± 0.4 vs 27.8 ± 0.7g p < 0.01; 20 weeks: 27.6 ± 0.5 vs 30.0 ± 0.9g p < 0.05) and as expected [24] an evident decrease at examination of fat pads volume. Despite these comparable blood pressure and plasma lipids levels and decreased fat mass, *Plin1*^*-/- *^*Ldlr*^-/- ^mice had after 10 weeks of atherogenic diet a trend for increase in atherosclerosis (figure 1) as quantified by the *en face *method. This increase was significant (+55%, p < 0.01) at 20 weeks. Figure 2 shows a representative sample of the extent of lesion in aortas from *Plin1*^*-/- *^*Ldlr*^-/- ^and *Ldlr*^-/- ^mice. Histological examination of plaques performed on aortas from mice fed since 20 weeks the atherogenic diet showed no differences in structure (elastic lamellae, fibrosis, cellularity) between *Plin1*^*-/- *^*Ldlr*^-/- ^and *Ldlr*^-/- ^mice (figure 3). The abundance of lymphocytes and macrophages estimated by immunocytology (figure 4) was comparable and there was no evidence of increased inflammation in plaques of *Plin1*^*-/- *^*Ldlr*^-/- ^mice. In addition we found no increase in the mRNA levels of MCP-1, IL-6 or IL1-β (data not shown) and only a non significant trend for higher values of TNFα mRNA (1.72 ± 0.37 10^-4 ^vs 1.13 ± 0.26 10^-5 ^ng/μg 18S RNA p = 0.10) in aortas from *Plin1*^*-/- *^*Ldlr*^-/- ^mice. The expression of SR-A, implicated in the uptake of modified lipoproteins, and of ABCA1 and ABCG1, controlling the efflux of cholesterol, were increased in aortas of *Plin1*^*-/- *^*Ldlr*^-/- ^mice (respectively 3.59 ± 1.06 10^-1 ^vs 4.81 ± 1.09 10^-2^, 5.85 ± 1.53 10^-3 ^vs 2.94 ± 1.03 10^-4 ^and 7.17 ± 2.95 10^-4 ^vs 1.44 ± 0.45 10^-4 ^ng/μg 18S RNA, p < 0.05 for all).

**Table 2:** Plasma lipid concentrations in mice after 10 or 20 weeks of atherogenic diet

	Total cholesterol g/l		Triacylglycerols g/l	
	***Ldlr***^***-/-***^	***Ldlr***^***-/- ***^***Plin1***^***-/-***^	***Ldlr***^***-/-***^	***Ldlr***^***-/- ***^***Plin1***^***-/-***^

10 weeks	9.99 ± 0.71	10.07 ± 0.96	1.32 ± 0.20	1.34 ± 0.55

20 weeks	8.56 ± 1.39	9.79 ± 0.32	0.75 ± 0.34	1.02 ± 0.38

Results are shown as mean and sem

## Discussion

The present results support an atheroprotective role of perilipin1 since the extent of atheroma lesions was increased in *Ldlr*^-/- ^mice with perilipin1 ablation. The overall histological appearance of the plaques was unchanged and we observed no increase in macrophages or lymphocytes infiltration, nor any significant increase in the mRNA concentrations of pro-inflammatory cytokines and chemokines. Therefore inflammation does not appear increased and the enhanced atheroma seems to result mainly form an increase in lipid deposits. Such an increase is demonstrated by the examination of aortas by the *en face *method since Red Sudan IV stains lipid deposits. An increased expression of SR-A could contribute to this increase in lipid deposits. The increase in ABCA1 and ABCG1 expression would on the contrary favour cholesterol efflux and could be an adaptative response to the increased accumulation of intra-cellular cholesterol. Perilipin1 can be found in macrophages and vascular smooth muscular cells and is present within atheroma plaques in foam cells originating from both cell types [18-22]. We used mice with a global invalidation of perilipin1 and cannot delineate the respective roles of macrophages and smooth muscular cells perilipin1 in the evolution of atheroma. This will need investigating mice with tissue specific invalidation. We cannot exclude an indirect effect on atheroma of perilipin1 ablation outside of the vasculature, i.e. in adipose tissue. However, blood pressure and plasma lipid levels were unaffected while body weight and fat mass (as appreciated by fat pads volume) were reduced by perilipin1 ablation. Therefore we can exclude a role for three major risk factors for atheroma (hypertension, higher plasma lipid levels, obesity) in the enhanced atherosclerosis we observed. Physical activity was also unchanged (data not shown) in agreement with previous reports [26]. Perilipin1 invalidation induces however some peripheral insulin-resistance, despite the reduced adiposity [27]. We cannot exclude a role for such insulin-resistance.

## Conclusion

In conclusion we found a protective role of perilipin1 against atherosclerosis. Evaluation of the respective roles of perilipin1 in macrophages and in smooth muscular cells and elucidation of the precise mechanisms implicated will require further studies.

## Competing interests

The authors declare that they have no competing interests.

## Authors' contributions

DL had in charge the mice strains and performed with JYL the quantification of atheroma and histological studies. FF and PDC had in charge mice strains and collected data, particularly plasma lipids and blood pressure. SN had in charge mRNA determinations. MB designed the study and wrote the manuscript. All authors participated in the analysis of data and the preparation of the manuscript.

## References

[B1] Ross R Cell biology of atherosclerosis Annu Rev Physiol 1995 57 791 804 10.1146/annurev.ph.57.030195.004043 7778883

[B2] De Winter M Hofker M Scavenging new insights into atherogenesis J Clin Invest 2000 105 1039 1041 10.1172/JCI9919 10772646 PMC300843

[B3] Cavelier CLI Rohrer L von Eckardstein A Lipid efflux by the ATP-binding cassette transporters ABCA1 and ABCG1 Biochim Biophys Acta 2006 176 655 666 10.1016/j.bbalip.2006.04.012 16798073

[B4] Chinetti G Gbaguidi F Griglio S Mallat Z Antonucci M Poulain P Chapman J Fruchart J Tedgui A Najib-Fruchart J CLA-1/SR-BI is expressed in atherosclerotic lesion macrophages and regulated by activators of peroxisome proliferator-activated receptors Circulation 2000 101 2411-2417 10.1161/01.cir.101.20.2411 10821819

[B5] Chen W Silver D Smith J Tall A Scavenger receptor-BI inhibits ATP-binding cassette transporter 1 mediated cholesterol efflux in macrophages J Biol Chem 2000 275 30794 30800 10896940 10.1074/jbc.M004552200

[B6] Liscum L Klansek J Nieman-Pick disease type C Curr Opin Lipidol 1998 9 131 135 9559270 10.1097/00041433-199804000-00009

[B7] Tabas I Consequences of cellular cholesterol accumulation: basic concepts and physiological implications J Clin Invest 2002 110 905 911 12370266 10.1172/JCI16452 PMC151158

[B8] Contreras J Lasuncion M Essential differences in cholesterol ester metabolism between human monocyte derived and J774 macrophages: evidence against the presence of HSL in human macrophage Arterioscler Thromb Vasc Biol 1993 14 443 452 10.1161/01.atv.14.3.443 8123650

[B9] Ghosh S Cholesteryl ester hydrolase in human monocyte/macrophage: cloning, sequencing, and expression of full length cDNA Physiol gernomics 2000 2 1 8 10.1152/physiolgenomics.2000.2.1.1 11015575

[B10] Khoo j Reue K Steinberg D Schotz M Expression of HSL mRNA in macrophages J Lipid Res 1993 34 1969 1974 8263420

[B11] Kimmel AR Brasaemle DL McAndrews-Hill M Sztalryd C C L Adoption of PERILIPIN as a unifying nomenclature for the mammalian PAT-family of intracellular lipid storage droplet proteins J Lipid Res 2010 51 468 471 10.1194/jlr.R000034 19638644 PMC2817576

[B12] Heid H Moll R Schweltick I Rackwitz H Keenan T Adipophilin is a specific marker of lipid accumulation in diverse cell types ans diseases Cell Tissue res 1998 294 309 321 10.1007/s004410051181 9799447

[B13] Buechler C Ritter M Duong C Orso E Kapinsky M Schmitz G Adipophilin is a sensitive marker for lipid loading in human blood monocytes Biochim Biophys Acta 2001 1532 97 104 11420178 10.1016/s1388-1981(01)00121-4

[B14] Wang X Reape T Li X Rayner K Webb C Burnand H Lysko P Induced expression of adipophilin mRNA in human macrophages stimulated with oxidized low-density lipoprotein and in atherosclerotic lesions FEBS Letters 1999 462 145 150 10.1016/S0014-5793(99)01521-5 10580108

[B15] Larigauderie G Furman C Jaye M Lasselin C Copin C Fruchart J Castro G Rouis M Adipophilin enhances lipid accumulation and prevents lipid efflux from THP-1macrophages: potential role in atherogenesis Arterioscler Thromb Vasc Biol 2004 24 504 510 10.1161/01.ATV.0000115638.27381.97 14707038

[B16] Paul A Chang BH-J Li L Yechoor VK Chan L Deficiency of Adipose Differentiation-Related Protein Impairs Foam Cell Formation and Protects Against Atherosclerosis Circulation Research 2008 102 12 1492 1501 10.1161/CIRCRESAHA.107.168070 18483409 PMC2773502

[B17] Lu XG-GJ Copeland NG Gilbert DJ Jenkins NA Londos C Kimmel AR The murine perilipin gene: the lipid droplet-associated perilipins derive from tissue-specific, mRNA splice variants and define a gene family of ancient origin Mamm Genome 2001 12 741 749 10.1007/s00335-01-2055-5 11641724

[B18] Forcheron F Legedz L Chinetti G Feugier P Letexier D Bricca G Beylot M Genes of cholesterol metabolism in human atheroma: overexpression of perilipin and genes promoting cholesterol storage and repression of ABCA1 expression Arterioscler Thromb Vasc Biol 2005 25 1711 1717 10.1161/01.ATV.0000174123.19103.52 15961705

[B19] Hofnagel OBI Schnoor M Lorkowski S Robenek H Expression of perilipin isoforms in cell types involved in atherogenesis Atherosclerosis 2007 190 14 15 10.1016/j.atherosclerosis.2006.06.010 16842797

[B20] Larigauderie GBM Furman C Jaye M Fruchart JC Rouis M Perilipin, a potential substitute for adipophilin in triglyceride storage in human macrophages Atherosclerosis 2006 189 142 148 10.1016/j.atherosclerosis.2005.12.016 16442115

[B21] Persson JDE Nilsson J Lindholm MW Perilipin and adipophilin expression in lipid loaded macrophages Biochem Biophys Res Commun 2007 363 1020 1026 10.1016/j.bbrc.2007.09.074 17927964

[B22] Faber B KBJM C Niessen R Aarts P Boon W AS G Kitslaar P Tordoir J Daemen M Identification of genes potentially involved in rupture of human atherosclerotic plaques Circ Res 2001 89 547 554 10.1161/hh1801.096340 11557743

[B23] Yao P Tabas I Free cholesterol loading of macrophages induces apoptosis involving fas pathway J Biol Chem 2000 275 23807 23813 10.1074/jbc.M002087200 10791964

[B24] Martinez-Botas J Anderson J Tessier D Lapillonne A Chang B Quast M Gorenstein D Chen K L C Absence of perilipin results in leanness and reverses obesity in Lepr (db/db) mice Nat Genet 2000 26 474 479 10.1038/82630 11101849

[B25] D Langlois MH Bouazza L Parkalian A Sasaki T Bricca G JY Li Conditional inactivation of TGF-β type II receptor in smooth muscle cells and epicardium causes lethal aortic and cardiac defects Transgenic Res 2010 19 1069 1082 10.1007/s11248-010-9379-4 20213136

[B26] Tansey J Sztalryd C Gruia-Gray J Roush D Zee J Gavrilova O Reiman M Deng C Li C Kimmel A Perilipin ablation results in a lean mouse with aberrant adipocyte lipolysis, enhanced leptin production, and resistance to diet-induced obesity Proc Natl Acad Sci USA 2001 98 6494 6499 10.1073/pnas.101042998 11371650 PMC33496

[B27] Saha PK Kojima H Martinez-Botas J Sunehag AL Chan L Metabolic Adaptations in the Absence of Perilipin Journal of Biological Chemistry 2004 279 34 35150 35158 10.1074/jbc.M405499200 15197189

